# Dual‐Module Near‐Infrared Fluorophores Discovery System via Knowledge Transfer

**DOI:** 10.1002/advs.76196

**Published:** 2026-06-22

**Authors:** Yixin Zhu, Xia Ling, Xianhe Zhang, Chuanjiang Jian, Leilei Shi, Wentao Song, Xiaonan Wang, Bin Liu

**Affiliations:** ^1^ Department of Chemical and Biomolecular Engineering National University of Singapore Singapore Singapore; ^2^ School of Medicine Precision Research Center for Refractory Diseases in Shanghai General Hospital Shanghai Jiao Tong University Shanghai China; ^3^ Department of Chemical Engineering Tsinghua University Beijing China

**Keywords:** bioimaging, deep learning, molecular design, near‐infrared, optical properties

## Abstract

In vivo near‐infrared (NIR) imaging is an emerging technique in biomedical research. It is particularly important for examining living tissue owing to its deep tissue penetration and low autofluorescence within the NIR optical window. Here, we present a deep learning discovery system for suggesting potential NIR fluorophores. Our approach employs a dual‐module framework, incorporating a predictive module with transfer learning to estimate properties and a generative module for constructing synthetically accessible NIR fluorophore candidates. This system predicts key optical properties, addressing limitations of labor‐intensive experimentation, with transfer learning incorporated to handle data scarcity. Through this system, three molecules (NTDT‐TPA, NPA‐BTD, DPP‐TPA) are synthesized for experimental validation. Among them, NTDT‐TPA is formulated into nanoparticles and evaluated in vitro and in vivo, showing its potential for fluorescence bioimaging.

## Graphical abstract

This study presents a dual‐module deep learning system for the design of near‐infrared (NIR) fluorophores. A large molecular library is generated and analyzed, leading to the suggestions of promising candidates. The effectiveness of the system is further validated through the synthesis, characterization, and in vivo imaging, demonstrating its potential to accelerate the design and biomedical application of NIR fluorophores.

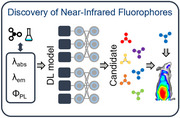

## 1 Introduction

Fluorophores are essential in many scientific and clinical research domains, including organic light‐emitting diodes, photodetectors, and bioimaging [1, 2, 3, 4, 5, 6]. These materials are specifically designed for various optical windows, covering different wavelength ranges, to suit application needs. Among them, the near‐infrared (NIR) optical window has attracted significant research interest. This is owing to the unique capabilities of organic molecules with NIR absorption or emission properties in providing real‐time insights into living biological tissues. The fundamental optical properties that define the performance of NIR fluorescent molecules are maximum absorption wavelength (λ_abs_), maximum emission wavelength (λ_em_), and photoluminescence quantum yield (Φ_PL_). These parameters together determine the performance of these molecules in bioimaging applications. Thus, a thorough understanding of these optical properties is key in guiding the design of high‐performance NIR fluorophores.

Significant research efforts have been focused on the exploration of NIR organic emissive dyes, with various strategies applied to investigate NIR fluorescent molecules [7, 8, 9]. A particularly promising approach centers on organic molecules constructed using an electron donor–acceptor (D–A) and donor–acceptor–donor (D–A–D) architecture [10, 11, 12]. This architecture facilitates the extension of emission wavelengths through the reduction of the bandgap, a consequence of intramolecular charge transfer between electron‐donating and electron‐accepting moieties [13]. One primary challenge in the development of efficient NIR materials lies in the scarcity of suitable acceptor units. The synthesis of electron‐deficient acceptor units, which are essential components of NIR fluorophores, presents significant challenges [14]. These difficulties are particularly marked in reactions such as bromination, where the inherent electron deficiency of these units complicates the process. Another important aspect in the context of NIR dyes is the Stokes shift. For bioimaging applications, NIR dyes with a substantial Stokes shift are preferred, as they effectively minimize interference from the excitation light in captured images, which reduces background noise and enhances image clarity [15]. Given these considerations, there is strong motivation to design or explore electron‐deficient units that can act as acceptors in NIR molecules. Alternatively, combining established donor and acceptor units could also yield molecules with a desirable Stokes shift.

Conventional methods for the design of NIR fluorescent molecules are labor‐intensive, involving time‐consuming manual alterations of molecular structures. These are subsequently verified through experimental protocols or theoretical computations, often utilizing time‐dependent density functional theory (TD‐DFT) [16, 17]. While TD‐DFT offers faster analyses, it is still limited by extensive computational costs and time, presenting a bottleneck in molecular design. Over years of research, these methods, despite their drawbacks, have led to the accumulation of a substantial body of data, comprising both theoretical and experimental results. This accumulated knowledge supports the adoption of deep learning in materials science. Deep learning, characterized by its ability to discern patterns and make accurate predictions, makes better use of existing data. Specifically, it facilitates the design of NIR fluorescent molecules with reduced reliance on experimental and computational resources, which streamlines the research process. This transition to deep learning marks a critical juncture in the field [18, 19, 20, 21, 22, 23, 24, 25, 26, 27, 28, 29, 30, 31]. Various studies have demonstrated progress in predictive analysis for fluorescent molecular properties. For example, Ju et al. utilized a machine learning approach with molecular fingerprints and models implemented in scikit‐learn to predict optical characteristics, including λ_abs_ and λ_em_ as well as Φ_PL_ [32]. In contrast, Joung et al. adopted a graph convolutional neural network model for predicting optical properties, demonstrating the diversity of computational strategies emerging in this field [33]. In addition, Souza et al. employed several machine learning algorithms and found that the Random Forest model performed best for predicting λ_em_ and Φ_PL_ [34]. Building on these studies, deep learning offers a practical route to accelerate the design of NIR fluorescent molecules.

**FIGURE 1:**
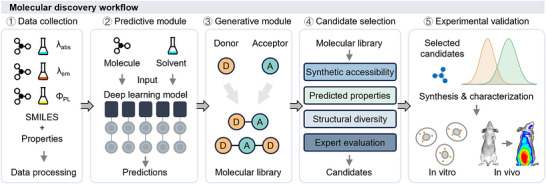
Overall workflow of the deep learning dual‐module framework for NIR fluorophore design. (1) Data collection of SMILES with solvent and photophysical labels (λ_abs_, λ_em_, Φ_PL_). (2) Predictive module with pretrain and molecule–solvent finetuning. (3) Generative module assembling donor–acceptor building blocks into D–A and D–A–D libraries. (4) Candidate selection by synthetic accessibility, predicted properties, structural diversity, and expert evaluation. (5) Experimental validation includes synthesis, characterization, and in vitro/in vivo evaluation.

In this study, we introduce an integrated dual‐module design system for molecular design, as shown in Figure 1. The system contains a predictive module and a generative module, specifically developed for the design of potential NIR fluorescent molecules. The predictive module, operationalized as a deep learning model, estimates critical optical properties, while the generative module constructs a diverse array of NIR fluorophore candidates. In more specific terms, first, recognizing the challenges posed by limited data, we incorporate transfer learning within the predictive module to enable accurate learning of optical properties from minimal datasets. In addition, our model accounts for fluorophore–solvent interactions, acknowledging the impact of the local molecular environment on the optical properties of NIR fluorescent molecules. Second, for the generative module, it is built upon and fully leverages expert knowledge in the field, while also possessing the ability to create molecular designs from structural units. This ensures both the synthetic accessibility and chemical validity of the generated molecules. Employing this strategy, the generative module produced a chemical library of 20,143 unique molecular structures. From this library, three structurally diverse molecules were selected based on predicted optical properties, synthetic accessibility, structural similarity, and expert judgment, and synthesized for experimental validation. We synthesized the selected molecules for experimental validation, and one of them was further formulated into nanoparticles and evaluated in vivo for fluorescence bioimaging. This study demonstrates that combining deep learning with expert knowledge can accelerate the design of NIR fluorescent materials.

## 2 Results

### 2.1 Prediction of Optical Properties

**FIGURE 2:**
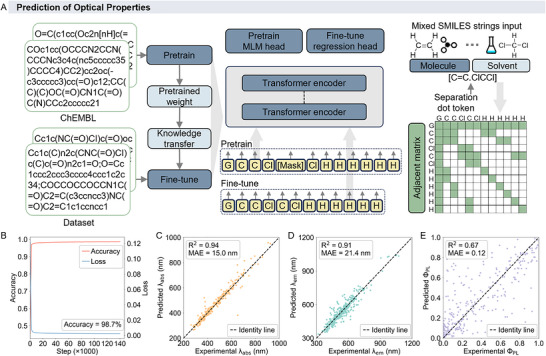
Schematic and performance of the predictive module. (A) Model architecture, pretraining on ChEMBL with masked language modeling, then knowledge transfer and finetuning on the photophysical dataset with molecule–solvent SMILES as input. (B) Pretraining accuracy and loss curves over training steps. (C–E) Overall prediction performance on the test set for (C) λ_abs_, (D) λ_em_, and (E) Φ_PL_.

NIR fluorophore data is scarce in the literature, which makes deep learning models hard to train. Considering this, we adopted a transfer learning architecture for the prediction of optical properties. Figure 2A shows the model architecture. The input is a Simplified Molecular‐Input Line‐Entry System (SMILES) string, and an adjacency matrix is used to restrict the attention mechanism to bonded atoms (Figure S1). Training follows a two‐stage approach. In the first stage, the model is pretrained on 1.0 million unlabeled structures extracted from the ChEMBL database [35] to learn general molecular representations that can be transferred to the downstream prediction task. Specifically, a masked‐atom language‐modeling task is used, where a subset of atom tokens in the SMILES is hidden, and the model is trained to recover them from chemical context [36]. The pretraining accuracy reached 98.7%, as shown in Figure 2B.

In the second stage, the model is fine‐tuned on the collected optical property dataset (λ_abs_, λ_em_, Φ_PL_), split 8:1:1 into training, validation, and test sets. The validation loss was monitored for early stopping to prevent overfitting. We first evaluated the model on two well‐known public fluorophore databases, ChemFluor [32] and Deep4Chem [37]. On ChemFluor (Figure S2), the mean absolute errors (MAEs) were 15.7 nm for λ_abs_, 18.7 nm for λ_em_, and 0.11 for Φ_PL_ (Figure S3 and Table S1). On Deep4Chem (Figure S4), the MAEs were 13.2 nm, 18.3 nm, and 0.11, respectively (Figure S5 and Table S2). These results are close to the baselines reported in the original studies. However, both public databases contain few NIR fluorophores. To improve coverage in the NIR region, we supplemented the ChemFluor dataset with additional NIR fluorophores collected from the literature (details in Methods). On the supplemented dataset (Figure S6), the MAEs on the test set were 15.0 nm for λ_abs_, 21.4 nm for λ_em_, and 0.12 for Φ_PL_ (Figure 2C–E and Table S3). Predicted and experimental values for fluorophores measured in multiple solvents show that our model captures solvent effects on optical properties (Tables S4–S6). However, adding NIR fluorophores dropped the overall prediction performance (Table S3). This reflects the difficulty of extending the model to a broader chemical space, a trend also reported when expanding fluorophore datasets [38]. The added NIR fluorophores extend coverage toward longer wavelengths, where predictions are harder due to extended conjugation and stronger solvent effects. In addition, Φ_PL_ is harder to predict than the wavelength properties, because non‐radiative decay pathways are difficult to capture from molecular structure alone [39].

### 2.2 Generation of Molecules

**FIGURE 3:**
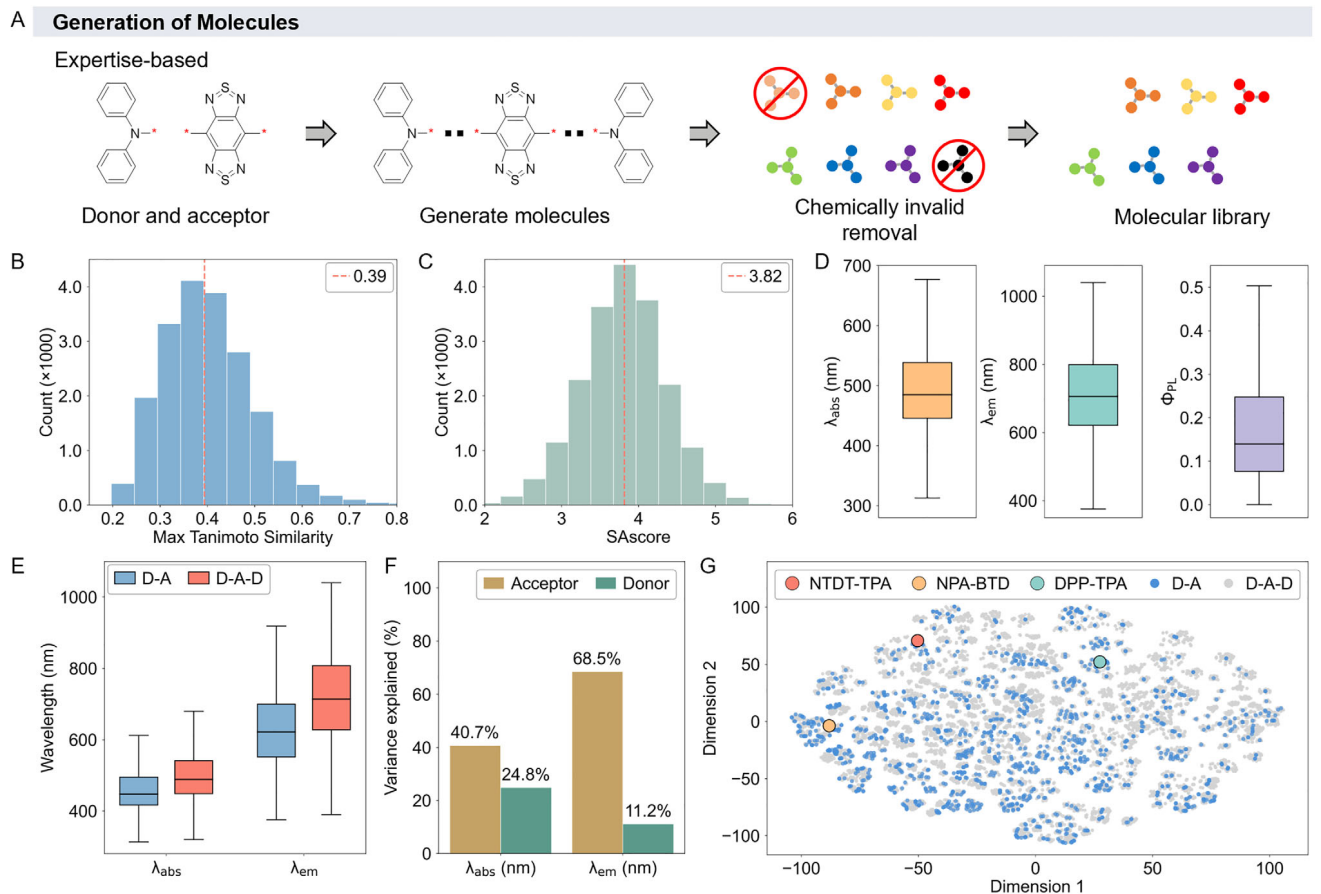
Generative module and characterization of the molecular library. (A) Expertise‐based combinatorial construction of donor and acceptor building blocks, chemical validity filtering, and construction of the molecular library. (B) Distribution of maximum Tanimoto similarity between generated and collected molecules. (C) Distribution of synthetic accessibility scores. (D) Distribution of predicted λ_abs_, λ_em_, and Φ_PL_ for the molecular library. (E) Distribution of predicted λ_abs_ and λ_em_ for D–A and D–A–D molecules in the molecular library. (F) Variance explained by acceptor and donor units for each property in D–A–D molecules. (G) t‐SNE visualization of the molecular library with three experimentally validated target molecules.

To ensure the chemical validity and synthetic accessibility of candidates, the method for molecular generation is structured around domain expert knowledge (Figure 3A). The most practical strategy for molecular design is to leverage chemical expertise. Forming D–A and D–A–D structured molecules has been shown to be an effective strategy to build NIR fluorescent molecules, as they consistently exhibit favorable optical properties in bioimaging. Therefore, the design of candidates in this work follows both the D–A and D–A–D structure to broaden the structural diversity of the generated library. We used 56 acceptor structures and 21 donor structures with different electronic strengths in this study (Tables S7 and S8), with slightly more acceptor structures owing to their greater synthetic difficulty. In addition, each donor and acceptor structure carries expert‐predefined sites that mark where couplings can occur. These annotations encode a simple reaction‐based connection rule set in the generative module, keeping bonds correct and removing unworkable connections. As a result, the generated structures stay within synthetically accessible regions of D–A and D–A–D space.

With the designed generative module and strategy, we generated a chemical library containing 20,143 candidates (technical details are described in the Methods section). To avoid the generation of chemically invalid molecular structures, a validity check was implemented using the RDKit built‐in checker [40]. We note that our generator enumerates combinations of known donor and acceptor units rather than generating scaffolds from scratch. This trades scaffold novelty for synthetic reliability. However, the generated library still covers chemical space that differs from the collected dataset. The maximum Tanimoto similarity between each generated molecule and the collected dataset has a median of 0.39 (Figure 3B). In addition to chemical validity, another decisive indicator for molecular generation is synthetic accessibility. Hence, to evaluate the synthetic feasibility of candidates in the chemical library, we employed the synthetic accessibility score (SAscore) [41]. All generated candidates in our library have SAscore below 6 (the commonly used synthetic accessibility threshold), with a median of 3.82 (Figure 3C), indicating that the expert‐predefined coupling rules effectively constrain generation within synthetically accessible space. It should be noted that SAscore evaluates synthetic complexity based on fragment frequency and does not directly reflect actual synthetic yields or purification difficulty. Here, we use it as a preliminary filter to exclude molecules that are likely to pose significant synthetic challenges.

### 2.3 Molecular Library Evaluation

The molecular library from the generative module was estimated by the trained predictive model to estimate the optical properties of each candidate. Figure 3D shows the distributions of predicted λ_abs_, λ_em_, and Φ_PL_ across the library. To guide molecule selection, we used predicted wavelengths as the main criterion, as the model shows relatively good agreement with experimental values. We first compared the optical properties across different molecular architectures. D–A–D molecules showed systematically longer predicted wavelengths than their D–A counterparts, motivating the use of D–A–D scaffolds for NIR coverage (Figure 3E). Within D–A–D molecules, the acceptor unit explained 40.7% and 68.5% of the variance in λ_abs_ and λ_em_, respectively, compared to 24.8% and 11.2% from the donor, indicating that the acceptor affects the emission wavelength more obviously in a D–A–D system (Figure 3F) [42]. Building on these trends, we designed a selection strategy through expert judgment, considering predicted wavelengths, SAscore, and structural similarity among candidates. Since the acceptor largely determines the wavelengths, we prioritized selecting acceptor units from different structural classes to maximize structural diversity. The donor was chosen to complement each acceptor, balancing predicted wavelength and synthetic feasibility. Φ_PL_ was used only as a reference due to larger prediction uncertainty. This strategy finally led to the selection of three molecules predicted to exhibit λ_em_ values above 700 nm, within the NIR window [43], for subsequent experimental validation: 4,4'‐(dithieno[3',2':5,6;2'',3'':7,8]naphtho[2,3‐c][1,2,5]thiadiazole‐2,5‐diyl)bis(N,N‐bis(4‐methoxyphenyl)aniline) (NTDT‐TPA), 4,7‐bis(4‐(naphthalen‐1‐yl(phenyl)amino)phenyl)benzo[c][1,2,5]thiadiazole‐5,6‐dicarbonitrile (NPA‐BTD), and 3,6‐bis(5‐(4‐(diphenylamino)phenyl)thiophen‐2‐yl)‐2,5‐bis(2‐ethylhexyl)‐2,5‐dihydropyrrolo[3,4‐c]pyrrole‐1,4‐dione (DPP‐TPA). Figure 3G shows their positions in the molecular library visualized by t‐distributed stochastic neighbor embedding (t‐SNE) [44]. They use fused dithieno[3',2':5,6;2'',3'':7,8]naphtho[2,3‐c][1,2,5]thiadiazole, fused benzo[c][1,2,5]thiadiazole‐5,6‐dicarbonitrile, and diketopyrrolopyrrole acceptors, respectively, and cover diverse structures.

### 2.4 Experimental Validation

**FIGURE 4:**
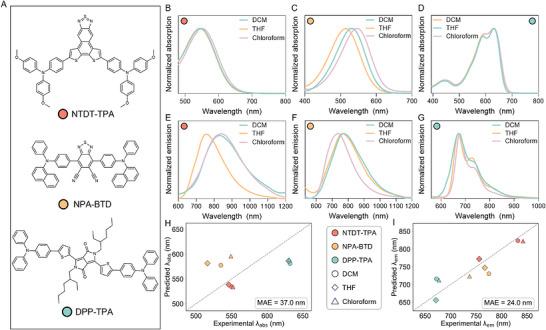
Chemical structures and photophysical properties of NTDT‐TPA, NPA‐BTD, and DPP‐TPA. (A) Chemical structures of NTDT‐TPA, NPA‐BTD, and DPP‐TPA; (B–G) Normalized absorption (B C D) and emission (E F G) spectra of NTDT‐TPA (B E), NPA‐BTD (C F), and DPP‐TPA (D G) in DCM, THF, and chloroform. (H–I) Predicted and experimental wavelengths of absorption (H) and emission (I) for NTDT‐TPA, NPA‐BTD, and DPP‐TPA in three solvents (DCM, THF, chloroform).

The chemical structures of the three selected molecules, NTDT‐TPA, NPA‐BTD, and DPP‐TPA, are shown in Figure 4A. Detailed synthetic procedures and structural characterization (^1^H NMR and ^13^C NMR) are provided in the supplementary materials (Figures S7‐S12). The absorption and emission spectra of the three molecules were measured in dichloromethane (DCM), tetrahydrofuran (THF), and chloroform (Figure 4B–G and Table S9). NTDT‐TPA, NPA‐BTD, and DPP‐TPA exhibited visible light absorption of 550, 535, and 632 nm with high molar absorption coefficients of 11400, 16800, and 81400 Lmol^−1^cm^−1^, and emission of 832, 775, and 672 nm, respectively, in DCM. NTDT‐TPA and NPA‐BTD are typical NIR fluorophores with maximum emission wavelengths above 700 nm. Although the maximum emission wavelength of DPP‐TPA is below 700 nm, its emission tail extends to 900 nm, indicating its NIR emission characteristics. Predicted and experimental λ_abs_ and λ_em_ values show that the model generally distinguished longer‐wavelength candidates from shorter‐wavelength ones (Figure 4H,I), though the agreement was less precise than that observed on the test set, which is expected when extrapolating to structures outside the training domain. Among the three NIR molecules, NTDT‐TPA exhibited the most red‐shifted emission, with a Φ_PL_ of 0.02 and good photostability in DCM (Figures S13 and S14). The fused dithienonaphthothiadiazole was a relatively weak acceptor with a band gap of around 2.6 eV [45, 46]. However, after coupling with donor units, the emission peak was predicted to shift beyond 800 nm, suggesting a large Stokes shift. This observation is notable as it indicates a substantial Stokes shift, which drew our research interest in this acceptor unit. A large Stokes shift is highly advantageous in bioimaging applications, as the background caused by excitation light can be excluded. While the extent of this benefit may vary depending on the excitation power, the presence of a large Stokes shift generally ensures clearer and more distinct imaging results, making it a preferred characteristic in bioimaging. Given this favorable property, we further selected NTDT‐TPA for fluorescence bioimaging.

**FIGURE 5:**
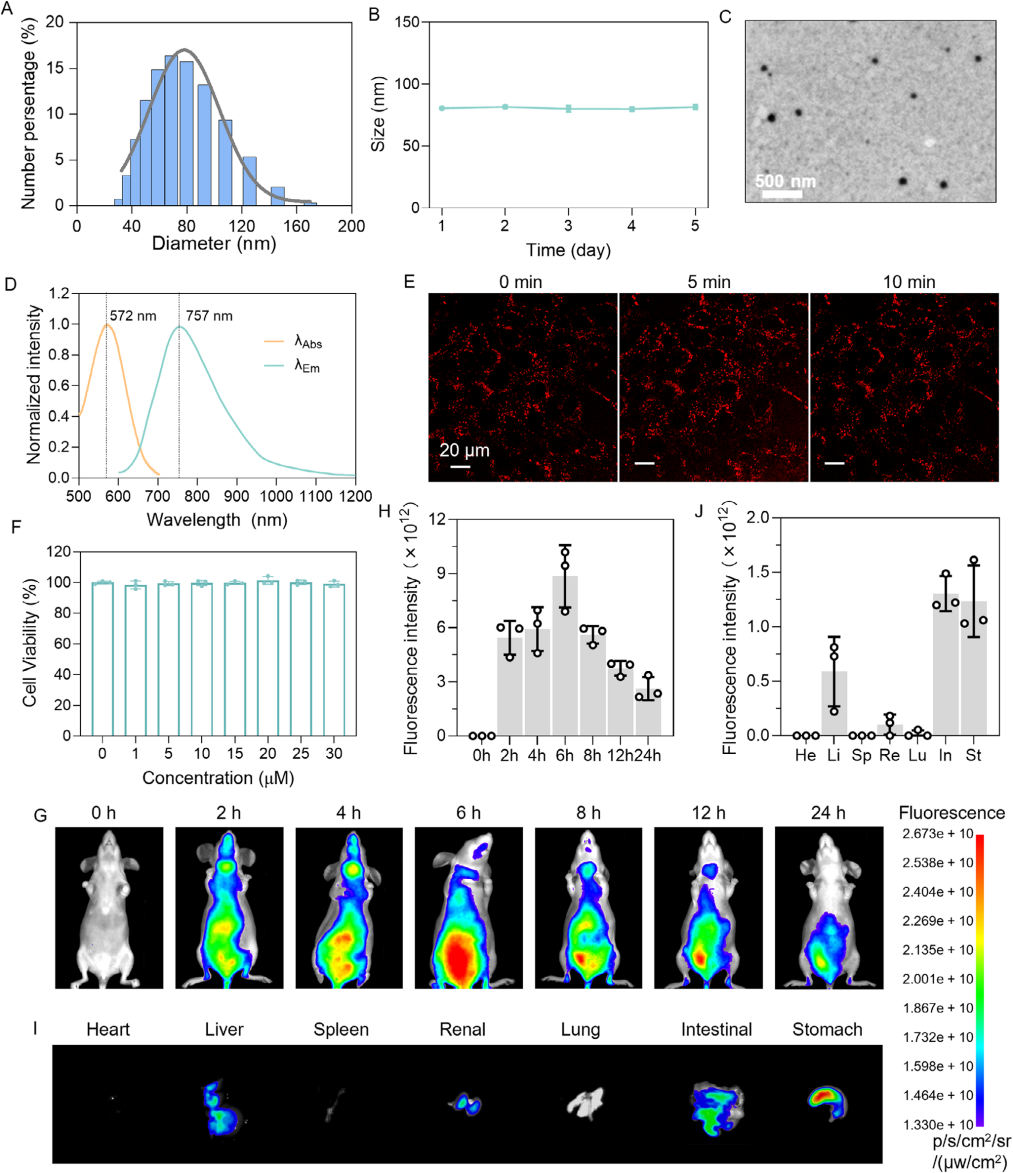
Characterization and in vivo imaging of NTDT‐TPA NPs. (A) Dynamic light scattering (DLS) analysis: correlation between nanoparticle number and size distribution. (B) Hydrodynamic diametersof NTDT‐TPA NPs among five days. (C) Transmission electron microscopy (TEM) image of nanoparticles. (D) Normalized absorption and emission spectra for NTDT‐TPA NPs. (E) Fluorescence imaging of cells treated with NTDT‐TPA NPs under light irradiation for 10 min. (F) Viability of cells treated with NTDT‐TPA NPs. (G) Representative time‐dependent in vivo fluorescence imaging of mice after intravenous injection of NTDT‐TPA NPs (500 µg/mL). (H) Time‐dependent fluorescence intensity of mice after intravenous injection of NTDT‐TPA NPs, (*n* = 3 independent biological samples, means ± SD). (I) Representative fluorescence imaging of organs (Heart, Liver, Spleen, Renal, Lung, Intestinal, and Stomach) excised from mice after 24 h intravenous post‐injection of NTDT‐TPA NPs. (J) Fluorescence intensity of organs excised from mice after intravenous injection of NTDT‐TPA NPs, (*n* = 3 independent biological samples, means ± SD).

To enable in vivo bioimaging, we encapsulated NTDT‐TPA into nanoparticles to improve water dispersibility. NTDT‐TPA‐based nanoparticles (NPs) were then fabricated with DSPE‐PEG 2K, and the procedure was detailed in the Supplementary Materials. The dynamic light scattering (DLS) measurement indicated that the average diameter of NPs was about 80 nm, which kept good stability among five days (Figure 5A,B). Its nano size was further verified by the transmission electron microscopy (TEM) images, demonstrating that its size is suitable for cell uptake and bioimaging (Figure 5C). We also measured the optical spectra of NTDT‐TPA NPs, which showed λ_abs_ and λ_em_ at 572 and 757 nm, respectively (Figure 5D). Next, the in vitro and in vivo fluorescence imaging performances of NTDT‐TPA NPs were evaluated. The cell uptake of NTDT‐TPA NPs was investigated by confocal laser scanning microscopy (CLSM). NIR fluorescence signal was observed after 12 h incubation (Figure 5E and Figure S15) and kept stable under 10 min laser scanning, indicating efficient internalization and good photostability of NTDT‐TPA NPs. The high cellular viability of NTDT‐TPA NPs demonstrated their good biocompatibility for fluorescence imaging applications (Figure 5F). The in vivo study was designed to assess the efficacy of NTDT‐TPA NPs in the fluorescence bioimaging of mice. Following the intravenous administration of NTDT‐TPA NPs, longitudinal fluorescence imaging was performed at sequential time points: immediately after injection (0 h) and post‐injection at 2, 4, 6, 8, 12, and 24 h. Critical observation was the peak fluorescence intensity at the 6‐h post‐injection (Figure 5G,H), highlighting the optimal temporal window for imaging post NP administration. After this zenith, a slow and steady reduction in fluorescence intensity was noted, elucidating the transient nature of the nanoparticle‐associated fluorescent signal within the systemic circulation. Further insightful data were gathered from the organ‐specific fluorescence imaging, depicted in Figure 5I,J. It clearly demonstrates the delivery of the nanoparticles to different organs and their deep tissue penetration capabilities. This in vivo study substantiates the viability of using NTDT‐TPA NPs for fluorescence bioimaging applications. The nanoparticles demonstrate effective circulatory distribution and adequate temporal stability for imaging purposes, along with a tendency for accumulation in different organs, which augments their suitability for targeted biological investigations.

## 3 Conclusion

In this study, we presented a deep learning‐based dual‐module molecular design system for NIR fluorescent molecules. The system was built with chemical expertise, so that the generated molecules are chemically valid and synthetically accessible. The predictive module estimates optical properties relevant to bioimaging, namely λ_abs_, λ_em_, and Φ_PL_. On the test set, predictions for the two wavelengths agreed with experimental measurements, while Φ_PL_ prediction showed larger deviations due to the difficulty of capturing non‐radiative decay pathways from molecular structure alone. Three molecules (NTDT‐TPA, NPA‐BTD, and DPP‐TPA) covering different acceptor classes and architectures were synthesized and characterized. Two molecules exhibited maximum emission wavelengths above 700 nm, while DPP‐TPA showed a maximum emission below 700 nm with an extended NIR tail. NTDT‐TPA was further formulated into nanoparticles and evaluated in a mouse model for fluorescence bioimaging. Furthermore, to make the predictive model accessible to non‐computational users, we developed a graphical user interface. It takes SMILES strings of the fluorophore as input and returns the predicted optical properties in multiple solvents (Figure S16).

Several limitations of the current work point to directions for future research. (i) The prediction accuracy decreased when the model was applied to generated molecules, with overall MAEs of 37.0 nm for λ_abs_ and 24.0 nm for λ_em_ across the three synthesized molecules and three solvents. This issue is especially relevant for candidates close to the NIR boundary. For example, DPP‐TPA showed an extended NIR tail, but its maximum emission remained below 700 nm, indicating that boundary predictions should be interpreted cautiously and confirmed experimentally. Expanding the training set or incorporating domain adaptation techniques could help address this limitation. (ii) Φ_PL_ remains harder to predict than wavelength properties, because structure‐based inputs do not fully describe non‐radiative emission loss. Future models may benefit from additional information related to molecular rigidity and excited‐state relaxation. (iii) Although the model captures general solvent‐dependent trends through molecule–solvent SMILES input, it does not explicitly model detailed solvent response, aggregation state, or biological microenvironment effects. Incorporating environment‐aware descriptors or condensed‐phase information may improve predictions under practical conditions. (iv) Application‐level properties such as photostability, aqueous solubility, biocompatibility, biodistribution, and clearance are not currently predicted by the model and still require experimental validation. Including standardized data for these properties would make the system more comprehensive for bioimaging applications. Overall, this work demonstrates that combining deep learning with chemical expertise can accelerate the design of NIR fluorescent molecules for bioimaging. The system does not replace traditional experimental methods but serves as a pre‐screening step to speed up synthesis decisions.

## 4 Methods

### 4.1 Data Collection and Processing

For pretraining, we used the ChEMBL database as the source of unlabeled molecular structures. Multi‐fragment entries were removed, and all SMILES were canonicalized using RDKit. 1.0 million structures were randomly sampled for the masked‐atom language‐modeling task. For fine‐tuning, we adopted the ChemFluor database as our primary data source, as it focuses specifically on fluorophores with λ_abs_, λ_em_, and Φ_PL_ measurements across multiple solvents. The Deep4Chem database was also processed using the same pipeline. Building upon ChemFluor, we supplemented with 181 additional NIR fluorophore entries collected from the literature, resulting in 2,944 unique molecules and a total of 4329 fluorophore–solvent combinations. All molecular data were processed and standardized using RDKit. SMILES strings for both fluorophores and solvents were canonicalized to ensure consistent molecular representations. Entries containing salt or mixtures were removed. For the optical properties, when the same fluorophore–solvent pair appeared more than once, we checked whether the reported values were consistent (within 10 nm for wavelengths and 0.1 for Φ_PL_). Consistent duplicates were averaged, and inconsistent ones were discarded. In the ChemFluor and Deep4Chem datasets, solvents with fewer than 10 entries were excluded to avoid unreliable predictions from underrepresented solvents.

### 4.2 Predictive Module

The deep learning model in the predictive module was trained using the dataset collected from the literature in this work. The predictive model is designed based on the bidirectional encoder representations from the transformers (BERT) architecture, combined with concepts from the graph neural network (GNN) [47, 48, 49]. The model accepts SMILES strings of molecules and solvents as input, where a dot token is used to merge them. For example, ethene in dichloromethane is represented as [C = C.ClCCl]. The architecture comprises an embedding layer, a transformer layer, and an output head. First, atom tokens are transformed into a distributional space. The transformer layer then applies an attention mechanism for node information aggregation, enhanced with multi‐head attention, residual connections, and layer normalization. Finally, an output head processes the output of the transformer layers to generate the target predictions (λ_abs_, λ_em_, and Φ_PL_ in this case). During the training stage, the loss function was defined as the mean squared error (MSE), calculated as the average of the squared differences between the actual values and the predicted values across all data points. Detailed model parameters are listed in Table S10.

### 4.3 Generative Module

The creation of a chemical library requires an expertise‐based approach. This process began with the careful selection of unique acceptor and donor molecular structures with distinct characteristics influencing optical properties. These selections, made by our NIR specialists, focused on structures with predefined points, critical for subsequent molecular modifications. Utilizing RDKit, we transformed these structures from standard SMILES notation into Mol objects. One example involves the representation of the fused dithienonaphthothiadiazole acceptor unit in the synthesized NIR fluorescent molecule, denoted as [*c1cc2c3cc4c(cc3c3cc(*)sc3c2s1)N = S = N4]. Within this notation, asterisks identify the predefined points for the incorporation of additional functional units. This strategy included the systematic enumeration of both acceptor and donor structures, with the specific intention of generating candidates that conform to both the D–A and D–A–D molecular frameworks. Each molecule was constructed step by step, incorporating single bonds one at a time. We configured our generative model to first construct D–A structures and then extend them into both symmetric and asymmetric D–A–D structures by coupling a second donor unit. This step was augmented with a validation protocol, wherein each generated structure was cross‐referenced against our dataset to confirm uniqueness and avoid redundant data. Every stage of the generation process was recorded, providing an explicit account of the synthetic evolution of each potential NIR fluorescent molecule from its original acceptor and donor units.

### 4.4 Visualization of the Chemical Library

To visualize the chemical space of the generated library, we computed Morgan fingerprints for each molecule using RDKit, with a radius of 2 and 2048 bits. Morgan fingerprints encode the local chemical environment around each atom, providing a fixed‐length binary representation suitable for comparing molecular similarity. These high‐dimensional fingerprints were then projected into a two‐dimensional space using t‐SNE. t‐SNE preserves local neighborhood structure, allowing molecules with similar substructures to appear close together in the embedding. The resulting visualization shows the distribution of D–A and D–A–D candidates across the library, with the positions of the selected molecules highlighted to illustrate their coverage of different regions of the chemical space.

### 4.5 Animal Model

The experiment procedures involving animals were conducted following approval from the Animal Ethics Committee of Shenzhen TopBiotech Co., Ltd. (Approved number: TOP‐IACUC‐2022‐0198). BALB/c female mice aged 4 weeks were procured from Zhuhai Bestest Biotechnology Co., Ltd. NTDT‐TPA nanoparticles (500 µg/mL) were intravenously injected, and fluorescence images were acquired at 0, 2, 4, 6, 8, 12, and 24 h post‐injection by using AniView100.

## Author Contributions

**Chuanjiang Jian**: investigation, formal analysis, writing – review and editing. **Xiaonan Wang**: funding acquisition, project administration, writing – review and editing, supervision. **Yixin Zhu**: conceptualization, methodology, software, data curation, investigation, formal analysis, visualization, writing – original draft, writing – review and editing. **Leilei Shi**: investigation, formal analysis, writing – review and editing. **Xia Ling**: methodology, investigation, validation, formal analysis, visualization, writing – review and editing. **Xianhe Zhang**: conceptualization, methodology, data curation, investigation, validation, formal analysis, visualization, writing – original draft, writing – review and editing, software. **Bin Liu**: conceptualization, supervision, funding acquisition, project administration, resources, writing – review and editing. **Wentao Song**: investigation, formal analysis, writing – review and editing, data curation.

## Conflicts of Interest

The authors declare no conflicts of interest.

## Supporting information




**Supporting File**: advs76196‐sup‐0001‐SuppMat.docx.

## Data Availability

All data, code, and user interfaces generated and analyzed in this work can be accessed at https://github.com/zhuyixintc/NIR_Fluorophore_Design.
