# Supplementary material for: Dual‐Module Near‐Infrared Fluorophores Discovery System via Knowledge Transfer
Source: Adv Sci (Weinh). 2026 Jun 22;13(57):e76196. doi: 10.1002/advs.76196 (PMC13336510; doi:10.1002/advs.76196)
Supplement: Supplementary file 1 [file ADVS-13-e76196-s001.docx]

Supporting Information

Dual-Module Near-Infrared Fluorophores Discovery System via Knowledge Transfer

*Yixin Zhu^[a]‡^, Xia Ling ^[a]‡^, Xianhe Zhang^[a]^, Chuanjiang Jian^[b]^, Leilei Shi^[b]^, Wentao Song^[a]^, Xiaonan Wang^[c]*^, Bin Liu^[a]*^*

^[a]^ Y. Zhu, X. Ling, X. Zhang, W. Song, B. Liu

Department of Chemical and Biomolecular Engineering, National University of Singapore, Singapore 117585, Singapore

^[b]^ C. Jian, L. Shi

Precision Research Center for Refractory Diseases in Shanghai General Hospital, School of Medicine, Shanghai Jiao Tong University, Shanghai 200025, China

^[c]^ X. Wang

Department of Chemical Engineering, Tsinghua University, Beijing 100084, China

^*^Corresponding Author: Xiaonan Wang - wangxiaonan@tsinghua.edu.cn, Bin Liu - cheliub@nus.edu.sg

^‡^These authors contributed equally: Yixin Zhu, Xia Ling

**1. Materials and Methods**

**1.1. Synthesis of Selected Molecules**

The synthetic work was referred to *J. Mater. Chem. C*, 2014, 2, 3972 and *J. Mater. Chem. A*, 2015,3, 4229-4238 [1,2].

5,6-Di(thiophen-3-yl)benzo[c][1,2,5]thiadiazole (1): 5,6-Dibromo-2,1,3-benzothiadiazole (1 g, 3.40 mmol) and 3-thiopheneboronic acid (1.09 g, 8.51 mmol) were dissolved in dioxane (40 mL) and a solution of potassium carbonate (2.82 g, 20.40 mmol) in water (14 mL) was added. Ar was bubbled through the reaction mixture for 10 min, then tetrakis(triphenylphosphine)palladium (Pd(PPh_3_)_4_, 393 mg, 0.34 mmol) was added. The reaction mixture was heated at 100 ºC overnight. The mixture was cooled to room temperature, DCM (dichloromethane) was added after dioxane was evaporated, and the organic phase was washed three times with water. The organic layer was dried over Na_2_SO_4_, then the solvent was removed under reduced pressure. The crude product was purified by silica gel column chromatography (hexane : DCM = 2 : 1 as an eluent) to give a yellow solid with yield of 83%.

Dithieno[3'2':5,6;2'',3'':7,8]naphtho[2,3-c][1,2,5]thiadiazole (NTDT) (2): A solution of (1) (500 mg, 1.66 mmol) in DCM (100 mL) was bubbled with Ar for 15 min. A solution of iron(III) chloride (600 mg, 3.67 mmol) in nitromethane (15 mL) was bubbled with Ar for 15 min, followed by adding into solution (1). After a 2-hour reaction, MeOH (30 mL) was added and kept 30 min stirring. All the solvent was removed by evaporation under reduced pressure. The crude product was purified through recrystallization in acetone to give red powder with yield of 62%.

NTDT-2Br (3): To a solution of compound (2) (125 mg, 0.42 mmol) in chloroform (50 mL), NBS (164 mg, 0.92 mmol) was added, and the mixture was stirred at 70℃ for 12 h. After cooling down to room temperature, precipitates were filtered by a funnel. The product was purified through recrystallization in toluene, affording dark red powders.

NTDT-TPA (4): Compound (3) (80 mg, 0.17 mmol) and (4-(bis(4-methoxyphenyl)amino)phenyl)boronic acid (148 mg, 0.42 mmol) were dissolved in dioxane (30 mL), then a solution of potassium carbonate (140 mg, 1.02 mmol) in water (10 mL) was added. Ar was bubbled through the reaction mixture for 10 min, and tetrakis(triphenylphosphine)palladium (Pd(PPh_3_)_4_) (10 mg) was added. The reaction mixture was heated at 100 ºC overnight. The mixture was cooled to room temperature, DCM was added after evaporation of solvent. Following three times of extraction, the organic layer was dried over Na_2_SO_4_ and the solvent was removed under reduced pressure. The crude product was purified by silica gel column chromatography (DCM : hexane = 1 : 1) to give black solid with yield of 36%. ^1^H NMR (400 MHz, Chloroform-*d*) *δ* 8.85 (s, 2H), 7.96 (s, 2H), 7.53 (d, *J* = 7.3 Hz, 4H), 7.12 (d, *J* = 7.4 Hz, 8H), 6.98 (d, *J* = 7.4 Hz, 4H), 6.88 (d, *J* = 7.3 Hz, 8H), 3.83 (d, *J* = 1.9 Hz, 12H). ^13^C NMR (101 MHz, Chloroform-*d*) δ 156.34, 152.50, 149.01, 142.98, 140.50, 134.34, 131.57, 130.22, 127.08, 126.84, 125.54, 120.23, 117.33, 114.95, 114.80, 55.66.

NPA-BTD (5): (4-(Naphthalen-1-yl(phenyl)amino)phenyl)boronic acid (678 mg, 2 mmol) and 4,7-dibromobenzo[c][1,2,5]thiadiazole-5,6-dicarbonitrile (343 mg, 1 mmol) were dissolved in tetrahydrofuran (THF, 30 mL) and a solution of potassium carbonate (414 mg, 3 mmol) in water (7 mL) was added. Ar was bubbled through the reaction mixture for 10 min, then added tetrakis(triphenylphosphine)palladium (Pd(PPh_3_)_4_, 230 mg, 0.2 mmol). The reaction mixture was stirred under argon condition at 70 ºC for 4 days. The mixture was cooled to room temperature and added with water (100 mL), which was further extracted with DCM (50 mL) for 3 times. The organic layer was dried over Na_2_SO_4_ for 1 h, then the solvent was removed under reduced pressure to obtain crude product, which was purified by silica gel column chromatography (hexane/DCM = 1 : 1 as an eluent) to give NPA-BTD as a brown solid (30 mg, yield 4%). ^1^H NMR (400 MHz, Chloroform-*d*) *δ* 8.00 (d, *J* = 8.4 Hz, 2H), 7.92 (d, *J* = 7.6 Hz, 2H), 7.85 (d, *J* = 8.4 Hz, 2H), 7.65 (d, *J* = 9.4 Hz, 4H), 7.55-7.43 (m, 8H), 7.29-7.27 (m, 8H), 7.07 (d, *J* = 8.8 Hz, 6H). ^13^C NMR (101 MHz, Chloroform-*d*) δ 154.68, 150.79, 146.84, 142.40, 140.55, 135.46, 131.65, 131.39, 129.63, 128.67, 127.97, 127.59, 127.05, 126.57, 124.19, 124.10, 124.09, 123.96, 118.81, 116.35, 111.57.

DPP (6): 3,6-di(thiophen-2-yl)-2,5-dihydropyrrolo[3,4-c]pyrrole-1,4-dione (3 g, 10 mmol) and K_2_CO_3_ (2.76 g, 20 mmol) were dissolved in DMF (100 mL), which was added with 3-(bromomethyl)heptane (10 mL 40 mmol). The reaction was stirred at 140℃ under argon condition for 24 h. After cooling to room temperature, the reaction was concentrated in vacuo and purified with silica gel column chromatography (DCM/MeOH = 20:1 as eluent) to afford DPP as a red solid (300 mg, yield 6%).

DPP-TPA (7): DPP (6) (37 mg, 0.07 mmol), 4-bromo-N,N-diphenylaniline (100 mg, 0.28 mmol), palladium (II) acetate (10 mg, 0.04 mmol), and pivalic acid (2 μL) were dissolved in N-methyl-2-pyrrolidone (NMP, 2 mL). The reaction was stirred at 110℃ under argon atmosphere for 5 h. After cooling to room temperature, the reaction was concentrated in vacuo and purified with silica gel column chromatography (Hexane:DCM = 1:1 as eluent) to afford DPP-TPA as a purple solid (25 mg, yield 35%). ^1^H NMR (400 MHz, Chloroform-*d*): ẟ 8.98 (d, *J* = 4.0 Hz, 2H), 7.52 (d, *J* = 8.8 Hz, 4H), 7.37 (d, *J* = 4.0 Hz, 2H), 7.29 (t, *J*_1_ = 16.0 Hz, *J*_2_ = 8.4 Hz, 8H), 7.14 (d, *J* = 7.6 Hz, 8H), 7.10-7.06 (m, 8H), 4.08-4.05 (m, 4H), 1.94 (d, *J* = 6.4 Hz, 2 H), 1.39-1.25 (m, 16H), 0.93-0.84 (m, 12H); ^13^C NMR (101 MHz, Chloroform-*d*): ẟ 161.80, 149.77, 148.56, 147.14, 139.68, 137.03, 129.46, 127.90, 126.95, 126.67, 125.01, 123.69, 123.47, 122.85, 107.99, 46.00, 39.24, 30.36, 28.54, 23.72, 23.11, 14.07, 10.62.

**1.2. Photophysical properties characterization**

Absorption spectra of NTDT-TPA, NPA-BTD and DPP-TPA were measured in DCM, THF and chloroform by using Shimadzu UV-2600i spectrophotometer (Japan). The absorption spectra of NTDT-TPA in DCM upon light irradiation for 0-30 mins was collected to assess its photostability. NIR emission spectra of NTDT-TPA, NPA-BTD and DPP-TPA were measured in DCM, THF and chloroform by using Edinburgh Instruments FLS 1000.

**1.3. Fabrication of NTDT-TPA Nanoparticles**

NTDT-TPA (1 mg) in THF solution (0.5 mL) was poured into DSPE-PEG (2K) aqueous solution (10 mL, 1 mg/mL) and sonicated for 2 min. The organic solvent was removed by stirring the mixture at room temperature for 12 h to yield NPs. The hydrodynamic diameter of nanoparticles was measured by dynamic light scattering (DLS) using a NanoZS® instrument (Malvern Instruments, UK). The transmission electron microscopy (TEM) of the nanoparticle was recorded using Tecnai G2 F20.

**1.4.** **Cell culture**

4T1 cells were cultured in DMEM medium with 10% fetal bovine serum (FBS) and 1% penicillin-streptomycin at 37℃ in a humidity atmosphere with 5% CO_2_.

**1.5. Cell viability assessment**

4T1 cells were seeded into the 96-well plate with a density of 1X10^5^ cells/mL. After incubation for 12 h, NTDT-TPA NPs (20 μg/mL) were added and further incubated for 4 h. After continuing incubating for 24 h, DMEM solution of MTT (0.5 mg/mL, 100 μL) was added to each well. After incubation at 37℃ for 2 h, the MTT solution was removed and 100 μL DMSO was added into each well, and the plate was shaken for 10 min at room temperature to dissolve all the precipitates. The absorbance of the solution at 570 nm was measured by a microplate reader. Cell viability was calculated by the ratio of the absorbance of sample and control group.

**1.6. In vitro Photostability of NTDT-TPA NPs**

4T1 cells were seeded into the confocal dish with a density of 1X10^5^ cell/mL. After incubation for 12 h, NTDT-TPA NPs (20 μg/mL) were added and further incubated for 12 h. The fluorescent imaging photos were collected using confocal laser scanning microscope (CLSM). The cells were irradiated by using laser of CLSM for 0-10 min. For fluorescent imaging channel: λ_ex_ = 580 nm，λ_em_ = 600−750 nm, power: 20%, gain 1200. For light irradiation channel: λ_ex_ = 405 nm, power: 2%.

**1.7. Deep Learning Model**

The predictive model is constructed based on the bidirectional encoder representation from transformers (BERT) and graph neural networks (GNNs) [3,4]. It requires SMILES strings as the only input, which is a valued feature of our approach. An adjacency matrix was generated from the mixed SMILES strings and used to guide information exchange between chemically connected atoms. The adjacent matrix is used to describe molecular information, for example chemical bonds. The model has a two-stage training named pre-train stage and fine-tune stage.

In the pre-training stage, the masked language modelling (MLM) task is carried out. The MLM task can be considered as a fill-in-the-gap task. [MASK] tokens are used to replace a certain number of atoms in a molecule and the model is trained to fill those atoms replaced by the [MASK] tokens. Here the output layer is a classification layer. The masking percentage follows the setting in BERT, where 15% atoms will be randomly selected for masking. For each selected one, actions including replacing with [MASK] tokens, replacing with other atoms and keeping unchanged are performed with an 8:1:1 ratio. We first collected the SMILES strings of 2.0 million unlabeled structures extracted from ChEMBL database. Then, MLM training was carried out with randomly sampled 1.0 million data (90% training, 10% testing). We pre-trained the model for 10 epochs to learn molecular information in a large chemical space. Through this kind of unsupervised training of a large amount of unlabeled chemical structures, the model can develop knowledge and facilitate the property prediction task training in the fine-tune stage.

In fine-tune stage, the output layer switches to a prediction layer to carry out the regression task. We trained the downstream task targeting each optical property with the data set collected from literature (80% training, 10% validation, 10% testing). To better reflect the stability of the predictive model, we evaluated the predictive model 5 times. Our predictive model displayed well-training with no overfitting.

**2. Supporting Figures**

|  | G | C | C | Cl | C | Cl | H | H | H | H | H | H |
| --- | --- | --- | --- | --- | --- | --- | --- | --- | --- | --- | --- | --- |
| G | 1 | 1 | 1 | 1 | 1 | 1 | 1 | 1 | 1 | 1 | 1 | 1 |
| C | 1 | 1 | 1 | 0 | 0 | 0 | 1 | 1 | 0 | 0 | 0 | 0 |
| C | 1 | 1 | 1 | 0 | 0 | 0 | 0 | 0 | 1 | 1 | 0 | 0 |
| Cl | 1 | 0 | 0 | 1 | 1 | 0 | 0 | 0 | 0 | 0 | 0 | 0 |
| C | 1 | 0 | 0 | 1 | 1 | 1 | 0 | 0 | 0 | 0 | 1 | 1 |
| Cl | 1 | 0 | 0 | 0 | 1 | 1 | 0 | 0 | 0 | 0 | 0 | 0 |
| H | 1 | 1 | 0 | 0 | 0 | 0 | 1 | 0 | 0 | 0 | 0 | 0 |
| H | 1 | 1 | 0 | 0 | 0 | 0 | 0 | 1 | 0 | 0 | 0 | 0 |
| H | 1 | 0 | 1 | 0 | 0 | 0 | 0 | 0 | 1 | 0 | 0 | 0 |
| H | 1 | 0 | 1 | 0 | 0 | 0 | 0 | 0 | 0 | 1 | 0 | 0 |
| H | 1 | 0 | 0 | 0 | 1 | 0 | 0 | 0 | 0 | 0 | 1 | 0 |
| H | 1 | 0 | 0 | 0 | 1 | 0 | 0 | 0 | 0 | 0 | 0 | 1 |

**Figure S1.** Adjacent matrix of the predictive model. Ethene (molecule) and dichloromethane (solvent) are used below for illustration. G is the global token added to allow information exchange with other nodes.

**Figure S2.** Distribution of (A) λ_abs_, (B) λ_em_, and (C) Φ_PL_ in the ChemFluor dataset.

**Figure S3.** Prediction performance on the ChemFluor test set for (A) λ_abs_, (B) λ_em_, and (C) Φ_PL_. Scatter plots show predicted versus experimental values.

**Figure S4.** Distribution of (A) λ_abs_, (B) λ_em_, and (C) Φ_PL_ in the Deep4Chem dataset.

**Figure S5.** Prediction performance on the Deep4Chem test set for (A) λ_abs_, (B) λ_em_, and (C) Φ_PL_. Scatter plots show predicted versus experimental values.

**Figure S6.** Distribution of (A) λ_abs_, (B) λ_em_, and (C) Φ_PL_ in the supplemented dataset.

**Figure S7.** ^1^H NMR spectrum of NTDT-TPA.

**Figure S8.** ^13^C NMR spectrum of NTDT-TPA.

**Figure S9.** ^1^H NMR spectrum of NPA-BTD.

**Figure S10.** ^13^C NMR spectrum of NPA-BTD.

**Figure S11.** ^1^H NMR spectrum of DPP-TPA.

**Figure S12.** ^13^C NMR spectrum of DPP-TPA.

**Figure S13.** Spectra for measuring Φ_PL_ of NTDT-TPA in DCM.

**Figure S14.** (A) UV-Vis absorption of NTDT-TPA in DCM under white light irradiation for 30 min; (B) The plot of absorption intensity of NTDT-TPA in DCM at 550 nm under white light irradiation for 30 min. [NTDT-TPA] = 20 μM, light power: 150 mW/cm^2^.

**Figure S15.** Fluorescence imaging of cells treated with NTDT-TPA NPs under light irradiation for 0-10 min. [NTDT-TPA NPs] = 20 μg/mL.

**Figure S16.** Designed graphical user interface (GUI) for users without computation background to lower the technical barriers. Users can input the SMILES strings of a molecule; the SMILES string can be obtained by various methods such as ChemDraw and SciFinder^n^. By clicking the start button, users can get optical property prediction within a second. A scroll on the GUI is provided to access the optical properties across solvents.

**3. Supporting Tables**

**Table S1.** Model performance on the ChemFluor dataset (averaged across 5 runs).

|  | R^2^ | MAE | MSE | RMSE |
| --- | --- | --- | --- | --- |
| λ_abs_ | 0.93 | 15.7 | 615.4 | 24.8 |
| λ_em_ | 0.91 | 18.7 | 782.8 | 27.9 |
| Φ_PL_ | 0.68 | 0.11 | 0.03 | 0.17 |

**Table S2.** Model performance on the Deep4Chem dataset (averaged across 5 runs).

|  | R^2^ | MAE | MSE | RMSE |
| --- | --- | --- | --- | --- |
| λ_abs_ | 0.95 | 13.2 | 515.5 | 22.7 |
| λ_em_ | 0.90 | 18.3 | 814.2 | 28.5 |
| Φ_PL_ | 0.66 | 0.11 | 0.03 | 0.17 |

**Table S3.** Model performance on the supplemented dataset (averaged across 5 runs).

|  | R^2^ | MAE | MSE | RMSE |
| --- | --- | --- | --- | --- |
| λ_abs_ | 0.94 | 15.0 | 756.9 | 27.4 |
| λ_em_ | 0.91 | 21.4 | 1062.6 | 32.5 |
| Φ_PL_ | 0.67 | 0.12 | 0.03 | 0.18 |

**Table S4.** Predicted and experimental λ_abs_ for molecules measured in multiple solvents.

| SMILES | Solvent | Exp. | Pred. | Error |
| --- | --- | --- | --- | --- |
| CN(C)c1ccc2c(c1)C(C)(C)c1cc(C(=O)c3cn(C4CC(O)C(CO)O4)nn3)ccc1-2 | CS(C)=O | 405 | 406.9 | 1.9 |
| CN(C)c1ccc2c(c1)C(C)(C)c1cc(C(=O)c3cn(C4CC(O)C(CO)O4)nn3)ccc1-2 | CCO | 403 | 402.6 | 0.4 |
| CN(C)c1ccc2c(c1)C(C)(C)c1cc(C(=O)c3cn(C4CC(O)C(CO)O4)nn3)ccc1-2 | CC#N | 395 | 401.4 | 6.4 |
| Cc1ccc(-c2nc(N(C)C)sc2-c2ccc([N+](=O)[O-])s2)cc1 | CS(C)=O | 500 | 501.4 | 1.4 |
| Cc1ccc(-c2nc(N(C)C)sc2-c2ccc([N+](=O)[O-])s2)cc1 | ClCCl | 485 | 491.7 | 6.7 |
| Cc1ccc(-c2nc(N(C)C)sc2-c2ccc([N+](=O)[O-])s2)cc1 | CC#N | 483 | 486.2 | 3.2 |
| O=Cc1ccc2ccc3cccc4ccc1c2c34 | ClCCl | 392 | 387.8 | 4.2 |
| O=Cc1ccc2ccc3cccc4ccc1c2c34 | Cc1ccccc1 | 392 | 385.3 | 6.7 |
| O=Cc1ccc2ccc3cccc4ccc1c2c34 | CC#N | 389 | 380.5 | 8.5 |
| COC(=O)CN1C(=O)c2cc3ccc(N(C)C)cc3cc2C1=O | ClC(Cl)Cl | 380 | 372.3 | 7.7 |
| COC(=O)CN1C(=O)c2cc3ccc(N(C)C)cc3cc2C1=O | CC(C)=O | 375 | 377.9 | 2.9 |
| COC(=O)CN1C(=O)c2cc3ccc(N(C)C)cc3cc2C1=O | C1COCCO1 | 372 | 359 | 13 |
| [C-]#[N+]c1ccc(-c2ccc(N)cc2)cc1 | CS(C)=O | 318 | 316 | 2 |
| [C-]#[N+]c1ccc(-c2ccc(N)cc2)cc1 | CO | 308 | 305.5 | 2.5 |
| [C-]#[N+]c1ccc(-c2ccc(N)cc2)cc1 | ClC(Cl)Cl | 305 | 308.1 | 3.1 |
| CC(=O)c1ccc2c(c1)C(C)(C)c1cc(N(C)CC#Cc3cn([C@H]4C[C@H](O)[C@@H](CO)O4)c(=O)nc3N)ccc1-2 | CS(C)=O | 370 | 367.1 | 2.9 |
| CC(=O)c1ccc2c(c1)C(C)(C)c1cc(N(C)CC#Cc3cn([C@H]4C[C@H](O)[C@@H](CO)O4)c(=O)nc3N)ccc1-2 | CN(C)C=O | 366 | 357 | 9 |
| CCCCCCCCCCCCn1cc(C(=O)c2ccc3c(c2)C(C)(C)c2cc(N(C)C)ccc2-3)nn1 | Cc1ccccc1 | 399 | 397.6 | 1.4 |
| CCCCCCCCCCCCn1cc(C(=O)c2ccc3c(c2)C(C)(C)c2cc(N(C)C)ccc2-3)nn1 | C1COCCO1 | 393 | 395.6 | 2.6 |
| O=Cc1cc(N2CCCCC2)c2ccc3c(N4CCCCC4)cc(C=O)c4ccc1c2c43 | CC#N | 482 | 483.6 | 1.6 |
| O=Cc1cc(N2CCCCC2)c2ccc3c(N4CCCCC4)cc(C=O)c4ccc1c2c43 | CCOC(C)=O | 474 | 478.8 | 4.8 |
| O=Cc1cc(N2CCCCC2)c2ccc3c(C=O)cc(N4CCCCC4)c4ccc1c2c34 | CC#N | 490 | 491.3 | 1.3 |
| O=Cc1cc(N2CCCCC2)c2ccc3c(C=O)cc(N4CCCCC4)c4ccc1c2c34 | CO | 482 | 487.6 | 5.6 |
| [C-]#[N+]c1ccc(-c2ccc(N(C)C)cc2)cc1 | CN(C)C=O | 339 | 344.6 | 5.6 |
| [C-]#[N+]c1ccc(-c2ccc(N(C)C)cc2)cc1 | C1COCCO1 | 330 | 317.7 | 12.3 |

**Table S5.** Predicted and experimental λ_em_ for molecules measured in multiple solvents.

| SMILES | Solvent | Exp. | Pred. | Error |
| --- | --- | --- | --- | --- |
| O=Cc1cc(C=O)c2ccc3c(N4CCCCC4)cc(N4CCCCC4)c4ccc1c2c43 | CS(C)=O | 609 | 614.3 | 5.3 |
| O=Cc1cc(C=O)c2ccc3c(N4CCCCC4)cc(N4CCCCC4)c4ccc1c2c43 | ClCCl | 602 | 595.8 | 6.2 |
| O=Cc1cc(C=O)c2ccc3c(N4CCCCC4)cc(N4CCCCC4)c4ccc1c2c43 | CC(C)=O | 598 | 591.3 | 6.7 |
| O=Cc1cc(C=O)c2ccc3c(N4CCCCC4)cc(N4CCCCC4)c4ccc1c2c43 | CCCCCC | 557 | 554.9 | 2.1 |
| CCN(CC)c1nc(-c2ccccc2)c(-c2ccc([N+](=O)[O-])s2)s1 | C1CCOC1 | 609 | 584.6 | 24.4 |
| CCN(CC)c1nc(-c2ccccc2)c(-c2ccc([N+](=O)[O-])s2)s1 | Cc1ccccc1 | 580 | 557.7 | 22.3 |
| CCN(CC)c1nc(-c2ccccc2)c(-c2ccc([N+](=O)[O-])s2)s1 | CCCCCC | 537 | 544.4 | 7.4 |
| CCOC(=O)c1cc(C#Cc2ccc3ccccc3c2)c(C(=O)OCC)cc1C#Cc1ccccc1 | CS(C)=O | 446 | 448.3 | 2.3 |
| CCOC(=O)c1cc(C#Cc2ccc3ccccc3c2)c(C(=O)OCC)cc1C#Cc1ccccc1 | ClCCl | 427 | 427.1 | 0.1 |
| CCOC(=O)c1cc(C#Cc2ccc3ccccc3c2)c(C(=O)OCC)cc1C#Cc1ccccc1 | Cc1ccccc1 | 418 | 416.4 | 1.6 |
| O=Cc1cc(N2CCCCC2)c2ccc3c(C=O)cc(N4CCCCC4)c4ccc1c2c34 | CO | 626 | 679.2 | 53.2 |
| O=Cc1cc(N2CCCCC2)c2ccc3c(C=O)cc(N4CCCCC4)c4ccc1c2c34 | CCOC(C)=O | 576 | 605.9 | 29.9 |
| O=Cc1cc(N2CCCCC2)c2ccc3c(C=O)cc(N4CCCCC4)c4ccc1c2c34 | CCCCCC | 549 | 544.7 | 4.3 |
| CCN(CC)c1ccc(/C=C/C2=[O+][B-](F)(F)Oc3c2c(=O)oc2cc(N(CC)CC)ccc32)cc1 | CC#N | 644 | 649.2 | 5.2 |
| CCN(CC)c1ccc(/C=C/C2=[O+][B-](F)(F)Oc3c2c(=O)oc2cc(N(CC)CC)ccc32)cc1 | CCO | 635 | 651.8 | 16.8 |
| CCN(CC)c1ccc(/C=C/C2=[O+][B-](F)(F)Oc3c2c(=O)oc2cc(N(CC)CC)ccc32)cc1 | CCOC(C)=O | 617 | 625.4 | 8.4 |
| CCN(CC)c1ccc2c(c1)C(C)(C)c1cc(-c3oc4ccccc4c(=O)c3OC)ccc1-2 | CN(C)C=O | 595 | 547.5 | 47.5 |
| CCN(CC)c1ccc2c(c1)C(C)(C)c1cc(-c3oc4ccccc4c(=O)c3OC)ccc1-2 | CCOC(C)=O | 544 | 516.4 | 27.6 |
| CN(C)c1nc(-c2ccccc2)c(-c2ccc([N+](=O)[O-])o2)s1 | CC#N | 630 | 635.2 | 5.2 |
| CN(C)c1nc(-c2ccccc2)c(-c2ccc([N+](=O)[O-])o2)s1 | C1CCOC1 | 572 | 529.2 | 42.8 |
| CN(C)c1ccc(-c2ccc3c(=O)c(O)coc3c2)cc1 | CC#N | 559 | 498.3 | 60.7 |
| CN(C)c1ccc(-c2ccc3c(=O)c(O)coc3c2)cc1 | C1COCCO1 | 457 | 474.9 | 17.9 |
| CC1(C)OC(=C(C#N)C#N)C(S(=O)(=O)c2ccccc2)=C1/C=C/c1ccc(N(c2ccccc2)c2ccccc2)cc1 | CS(C)=O | 750 | 737.8 | 12.2 |
| CC1(C)OC(=C(C#N)C#N)C(S(=O)(=O)c2ccccc2)=C1/C=C/c1ccc(N(c2ccccc2)c2ccccc2)cc1 | Cc1ccccc1 | 650 | 648.5 | 1.5 |
| CC1(C)OC(=C(C#N)C#N)C(Sc2ccccc2)=C1/C=C/c1ccc(N(c2ccccc2)c2ccccc2)cc1 | ClCCl | 680 | 678.3 | 1.7 |
| CC1(C)OC(=C(C#N)C#N)C(Sc2ccccc2)=C1/C=C/c1ccc(N(c2ccccc2)c2ccccc2)cc1 | C1CCCCC1 | 550 | 542.1 | 7.9 |

**Table S6.** Predicted and experimental Φ_PL_ for molecules measured in multiple solvents.

| SMILES | Solvent | Exp. | Pred. | Error |
| --- | --- | --- | --- | --- |
| CC1(C)c2cc(C=O)ccc2-c2ccc(N3CCC3)cc21 | CS(C)=O | 0.84 | 0.7882 | 0.0518 |
| CC1(C)c2cc(C=O)ccc2-c2ccc(N3CCC3)cc21 | Cc1ccccc1 | 0.7 | 0.7272 | 0.0272 |
| CC1(C)c2cc(C=O)ccc2-c2ccc(N3CCC3)cc21 | C1COCCO1 | 0.66 | 0.6975 | 0.0375 |
| CN(C)c1ccc(C2=Nc3sc4cc(-c5cccs5)ccc4[n+]3[B-](F)(F)O2)cc1 | C1CCOC1 | 0.87 | 0.6919 | 0.1781 |
| CN(C)c1ccc(C2=Nc3sc4cc(-c5cccs5)ccc4[n+]3[B-](F)(F)O2)cc1 | Cc1ccccc1 | 0.84 | 0.8415 | 0.0015 |
| CN(C)c1ccc(C2=Nc3sc4cc(-c5cccs5)ccc4[n+]3[B-](F)(F)O2)cc1 | CC(C)=O | 0.65 | 0.4588 | 0.1912 |
| CCN(CC)c1ccc(-c2oc3ccccc3c(=O)c2OC)cc1 | CS(C)=O | 0.65 | 0.4185 | 0.2315 |
| CCN(CC)c1ccc(-c2oc3ccccc3c(=O)c2OC)cc1 | ClCCl | 0.63 | 0.4902 | 0.1398 |
| CCN(CC)c1ccc(-c2oc3ccccc3c(=O)c2OC)cc1 | O | 0.001 | 0.1044 | 0.1034 |
| CCN(CC)c1ccc2cc(-c3oc4ccccc4c(=O)c3OC)oc2c1 | CC(C)=O | 0.41 | 0.458 | 0.048 |
| CCN(CC)c1ccc2cc(-c3oc4ccccc4c(=O)c3OC)oc2c1 | CS(C)=O | 0.33 | 0.3567 | 0.0267 |
| CN(C)c1ccc(C2=Nc3sc4ccccc4[n+]3[B-](F)(F)O2)cc1 | Cc1ccccc1 | 0.84 | 0.8348 | 0.0052 |
| CN(C)c1ccc(C2=Nc3sc4ccccc4[n+]3[B-](F)(F)O2)cc1 | C1CCOC1 | 0.78 | 0.6479 | 0.1321 |
| CN(C)c1ccc2c(c1)C(C)(C)c1cc(-c3ccc4nsnc4c3)ccc1-2 | ClC(Cl)Cl | 0.04 | 0.1042 | 0.0642 |
| CN(C)c1ccc2c(c1)C(C)(C)c1cc(-c3ccc4nsnc4c3)ccc1-2 | O | 0.01 | 0.0546 | 0.0446 |
| O=Cc1cc(N2CCCCC2)c2ccc3c(N4CCCCC4)cc(C=O)c4ccc1c2c43 | CC(C)=O | 0.93 | 0.2644 | 0.6656 |
| O=Cc1cc(N2CCCCC2)c2ccc3c(N4CCCCC4)cc(C=O)c4ccc1c2c43 | CCO | 0.25 | 0.1864 | 0.0636 |
| CCN(CC)c1nc(-c2ccc(Br)cc2)c(-c2ccc([N+](=O)[O-])s2)s1 | CS(C)=O | 0.003 | 0.0157 | 0.0127 |
| CCN(CC)c1nc(-c2ccc(Br)cc2)c(-c2ccc([N+](=O)[O-])s2)s1 | CC#N | 0.0001 | 0.0037 | 0.0036 |
| O=[N+]([O-])c1ccc(-c2ccc(-c3ccc(-c4ccc([N+](=O)[O-])cc4)s3)s2)cc1 | Cc1ccccc1 | 0.22 | 0.5309 | 0.3109 |
| O=[N+]([O-])c1ccc(-c2ccc(-c3ccc(-c4ccc([N+](=O)[O-])cc4)s3)s2)cc1 | CS(C)=O | 0.09 | 0.2297 | 0.1397 |
| CCCCCCCCC(=O)c1ccc2c(c1)C(C)(C)c1cc(N(CC)CC)ccc1-2 | Cc1ccccc1 | 0.83 | 0.7654 | 0.0646 |
| CCCCCCCCC(=O)c1ccc2c(c1)C(C)(C)c1cc(N(CC)CC)ccc1-2 | CN(C)C=O | 0.75 | 0.6004 | 0.1496 |

**Table S7.** SMILES of acceptor structures with expert predefined points.

SMILES

*c1ccc(*)c2nsnc12

*c1c2c(c(*)c3nsnc13)N=S=N2

*c1c2nsnc2c(*)c2nc3c4ccccc4c4ccccc4c3nc12

*c1c2c(c(*)c3nn(*)nc13)N=S=N2

*c1c2nsnc2c(*)c2nc(-c3ccccc3)c(-c3ccccc3)nc12

*c1ccc(-c2sc(-c3ccc(*)s3)c3c2N=S=N3)s1

*c1cc2c(s1)c1sc(*)cc1c1c(*)c3c(c(*)c21)N=S=N3

*c1ccc(*)s1

*/C=c1/cn/c(=C\*)cn1

*c1ccc(*)c2c(*)cccc12

*C/C=C1\CCCc2c(-c3ccccc3)cc(-c3ccccc3)c(*)c21

*C/C=C1\CCCc2cccc(*)c21

*/C=c1\cc/c(=C2/C3=C(C(=O)N2C)/C(=c2/cc/c(=C\*)s2)N(C)C3=O)s1

*C1=CC2=Nc3cc(*)cn3C(F)(F)C2=C1

*C1=CC2=Cc3cc(*)cn3C(F)(F)C2=C1

*c1ccc2c(c1)C(F)(F)n1cc3c4n(cc3c1=C2C#N)C(F)(F)c1cc(*)ccc1C=4C#N

*C1=CC2C(=C(C#N)C#N)c3ccc(*)cc3C(=C(C#N)C#N)C2C=C1

*c1scc2c1c1cc3c(cc1c1c(*)sc(*)c21)N=S=N3

*c1ccc2c(c1)C(=C(C#N)C#N)c1cc(*)ccc1-2

*C1=C2C(=O)N(C)C(*)=C2C(=O)N1C

*c1ccc2c(ccc3nc4cc(*)ccc4nc32)c1

*c1cc2c(s1)/C(=C1\C(=O)Oc3cc4c(cc31)OC(=O)/C4=C1/C(=O)N(C)c3cc(*)sc31)C(=O)N2C

*c1cnc(*)c2nsnc12

*c1c(F)c(F)c(*)c2nsnc12

*c1c(C#N)c(C#N)c(*)c2nsnc12

*c1c2c(c(*)c3nsnc13)C(=O)N(C)C2=O

*c1c2nsnc2c(*)c2nc3c(nc12)-c1cccc2cccc-3c12

*c1cc2c(c3nsnc13)c1c3nsnc3c(*)cc1n2C

*c1ccc(*)c2c1C(=O)N(C)C2=O

*c1cc2c(s1)c1c3nsnc3c3c4sc(*)cc4n(C)c3c1n2C

*c1cc2c(cc(*)c3nsnc32)c2nsnc12

*c1c(F)c(F)c(*)c2nn(C)nc12

*c1cc(=C(C#N)C#N)c(*)cc1=C(C#N)C#N

*c1ccc2c(n1)C(=C(C#N)C#N)c1nc(*)ccc1-2

*c1ccc2c(c1)C(=C(C#N)C#N)c1cc3c(cc1-2)C(=C(C#N)C#N)c1cc(*)ccc1-3

*c1ccc2c(c1)N(C)c1cc3c(cc1C2=C(C#N)C#N)N(C)c1cc(*)ccc1C3=C(C#N)C#N

*c1c2c(=O)n(C)c(=O)c2c(*)c2c(=O)n(C)c(=O)c12

*c1c2cc3c(=O)n(C)c(=O)c3cc2c(*)c2cc3c(=O)n(C)c(=O)c3cc12

*c1ccc(C2=c3cc4c(cc3N(C)C2=O)=C(c2ccc(*)cc2)C(=O)N4C)cc1

*c1c2c(c(*)c3nn(C)nc13)C(=O)N(C)C2=O

*c1ccc2c(c1)N(C)C(=O)/C2=C1/C(=O)N(C)c2cc(*)ccc21

*c1cc2c3c(c(*)cc4c3c1C(=O)N(C)C4=O)C(=O)N(C)C2=O

*c1cc2c3ccc4c5c(c(*)cc(c6ccc7c(c1C(=O)N(C)C7=O)c62)c53)C(=O)N(C)C4=O

*c1c2nc3c(nc2c(*)c2nc4c(nc12)-c1ccc2c5c(ccc-4c15)C(=O)N(C)C2=O)-c1ccc2c4c(ccc-3c14)C(=O)N(C)C2=O

*c1cc2c3c(c(*)cc4c3c1C(=O)N(c1ccncc1)C4=O)C(=O)N(c1ccncc1)C2=O

*c1cc2c3c(c(*)cc4c3c1C(=O)N(c1ccccc1)C4=O)C(=O)N(c1ccccc1)C2=O

*c1sc(*)c2c1C(=O)N(C)C2=O

*c1c2c(c(*)c3cscc13)C(=O)N(C)C2=O

*c1cc2c(=O)n(C)c(=O)c3cc(*)sc3c2s1

*c1sc(*)c2c1C(=O)c1cscc1C2=O

*c1c(F)c(F)c(*)c2nc(-c3ccccc3)c(-c3ccccc3)nc12

*c1cc2c3c(c1)C(F)(F)N(C)c1cc(*)cc(c1-3)C(F)(F)N2C

*c1cc2c3c(ccc4c5c(*)cc6c7c(ccc(c1c34)c75)C(=O)N(c1ccccc1)C6=O)C(=O)N(c1ccccc1)C2=O

*C1=C2C(=O)N(CC(CC)CCCC)C(*)=C2C(=O)N1CC(CC)CCCC

*c1ccc(-c2ccc(C#N)c(C#N)c2)s1

*c1cc(C#N)c(C#N)cc1*

**Table S8.** SMILES of donor structures with expert predefined points.

SMILES

*c1ccc(N(c2ccc(OC)cc2)c2ccc(OC)cc2)cc1

*c1ccc(N(c2ccccc2)c2cccc3ccccc23)cc1

*c1ccc(N(c2ccccc2)c2ccccc2)s1

*c1ccc(N(c2ccccc2)c2ccccc2)cc1

*c1ccc2c(c1)C(CCC(=O)OCCC(C)(C)C)(CCC(=O)OCCC(C)(C)C)c1cc(*)ccc1-2

*c1ccc2c(c1)C(CCCCCCCC)(CCCCCCCC)c1cc(*)ccc1-2

*C1N(C)c2ccccc2C1(C)C

*c1cc(OCCCCCCBr)c(*)c(OCCCCCCBr)c1

*c1ccc2c(c1)C(CCCCCCBr)(CCCCCCBr)c1cc(*)ccc1-2

*c1cc2c(-c3cccs3)c3sc(*)cc3c(-c3cccs3)c2s1

*c1ccc(C(=C(c2ccccc2)c2ccccc2)c2ccccc2)cc1

*c1ccc(N(c2ccccc2)c2ccc(C(=C(c3ccccc3)c3ccccc3)c3ccccc3)cc2)cc1

*c1ccc(*)s1

*c1cc2cc3sc(*)cc3cc2s1

*c1cc2c(s1)-c1sc(*)cc1C2(C)C

*c1cc2cc3c(OC)c4sc(*)cc4cc3c(OC)c2s1

*c1sc(*)c2c1OCCO2

*c1ccc(-c2cc3cccc4cc(-c5ccc(*)cc5)c5ccc2n5c34)cc1

*N(c1ccccc1)c1ccccc1

*c1ccc(-c2ccc(N(c3ccccc3)c3ccccc3)cc2)s1

*c1ccc(-c2ccc(N(c3ccc(OC)cc3)c3ccc(OC)cc3)cc2)s1

**Table S9.** Photophysical properties of NTDT-TPA, NPA-BTD, and DPP-TPA.

| Compound | Solvent | λ_abs_ (nm)  Exp. (Pred.) | λ_em_ (nm)  Exp. (Pred.) | Molar absorption coefficient  (Lmol^-1^cm^-1^) |
| --- | --- | --- | --- | --- |
| NTDT-TPA | DCM | 550 (534) | 832 (824) | 11400 |
|  | THF | 546 (539) | 756 (772) | 8400 |
|  | Chloroform | 552 (534) | 842 (823) | 9600 |
| NPA-BTD | DCM | 535 (577) | 775 (730) | 16800 |
|  | THF | 516 (582) | 767 (747) | 24700 |
|  | Chloroform | 549 (596) | 737 (723) | 13300 |
| DPP-TPA | DCM | 632 (581) | 672 (716) | 81400 |
|  | THF | 630 (586) | 671 (656) | 64600 |
|  | Chloroform | 631 (589) | 677 (712) | 67400 |

**Table S10.** The parameters of the deep learning model.

| Parameters | Value |
| --- | --- |
| Number of layers | 6 |
| Number of attention heads | 4 |
| Embedding dimension | 256 |
| Dropout rate | 0.1 |
| Learning rate | 1E-04 |
| Batch size | 32 |

**References**

1.	Q. Shuai, H. T. Black, A. Dadvand, and D. F. Perepichka, “Dithienonaphthothiadiazole Semiconductors: Synthesis, Properties, and Application to Ambipolar Field Effect Transistors,” *Journal of Materials Chemistry C* 2, no. 20 (2014): 3972-3979. <https://doi.org/10.1039/C4TC00094C>.

2.	T. Nakanishi, Y. Shirai, and L. Han, “Synthesis and Optical Properties of Photovoltaic Materials based on the Ambipolar Dithienonaphthothiadiazole Unit,” *Journal of Materials Chemistry A* 3, no. 8 (2015): 4229-4238. <https://doi.org/10.1039/C4TA05101G>.

3.	F. Scarselli, M. Gori, A. C. Tsoi, M. Hagenbuchner, and G. Monfardini, “The Graph Neural Network Model,” *IEEE Transactions on Neural Networks* 20, no. 1 (2009): 61-80. <https://doi.org/10.1109/TNN.2008.2005605>.

4.	X. Zhang, C. Wu, Z. Yang, et al., “MG-BERT: Leveraging Unsupervised Atomic Representation Learning for Molecular Property Prediction,” *Briefings in Bioinformatics* 22, no. 6 (2021): bbab152. <https://doi.org/10.1093/bib/bbab152>.
